# Lipid Profiling Reveals Lipidomic Signatures of Weight Loss Interventions

**DOI:** 10.3390/nu15071784

**Published:** 2023-04-06

**Authors:** Kaiqing Lin, Wei Cheng, Qiwei Shen, Hui Wang, Ruwen Wang, Shanshan Guo, Xianmin Wu, Wei Wu, Peijie Chen, Yongfei Wang, Hongying Ye, Qiongyue Zhang, Ru Wang

**Affiliations:** 1 School of Exercise and Health, Shanghai Frontiers Science Research Base of Exercise and Metabolic Health, Shanghai University of Sport, Shanghai 200438, China; 2 Department of Endocrinology, Yangpu Hospital, School of Medicine, Tongji University, Shanghai 200090, China; 3 Department of General Surgery, Huashan Hospital, Fudan University, Shanghai 200433, China; 4 Shanghai Key Laboratory of Metabolic Remodeling and Health, Institute of Metabolism & Integrative Biology, Fudan University, Shanghai 200433, China; 5 State Key Laboratory of Genetic Engineering, School of Life Sciences, Fudan University, Shanghai 200438, China; 6 Department of Endocrinology and Metabolism, Huashan Hospital, Fudan University, Shanghai 200433, China; 7 Department of Neurosurgery, Huashan Hospital, Fudan University, Shanghai 200433, China

**Keywords:** lipidomics, exercise, laparoscopic sleeve gastrectomy, cushing

## Abstract

Obesity is an epidemic all around the world. Weight loss interventions that are effective differ from each other with regard to various lipidomic responses. Here, we aimed to find lipidomic biomarkers that are related to beneficial changes in weight loss. We adopted an untargeted liquid chromatography with tandem mass spectrometry (LC-MS/MS) method to measure 953 lipid species for Exercise (exercise intervention cohort, N = 25), 1388 lipid species for LSG (laparoscopic sleeve gastrectomy cohort, N = 36), and 886 lipid species for Cushing (surgical removal of the ACTH-secreting pituitary adenomas cohort, N = 25). Overall, the total diacylglycerol (DG), triacylglycerol (TG), phosphatidylethanolamine (PE), phosphatidylinositol (PI), phosphatidylserine (PS), and sphingomyelin (SM) levels were associated with changes in BMI, glycated hemoglobin (HbA1c), triglyceride, and total cholesterol according to weight loss interventions. We found that 73 lipid species changed among the three weight loss interventions. We screened 13 lipid species with better predictive accuracy in diagnosing weight loss situations in either Exercise, LSG, or Cushing cohorts (AUROC > 0.7). More importantly, we identified three phosphatidylcholine (PC) lipid species, PC (14:0_18:3), PC (31:1), and PC (32:2) that were significantly associated with weight change in three studies. Our results highlight potential lipidomic biomarkers that, in the future, could be used in personalized approaches involving weight loss interventions.

## 1. Introduction

Obesity, considered as a worldwide epidemic, is defined as a disproportionate body weight for height with various consequences including hypertension, dyslipidemia, cardiovascular disease (CVD), stroke, type 2 diabetes mellitus (T2DM), and premature mortality [1,2,3,4]. The Global Burden of Disease Obesity Collaborators reported that over 603.7 million adults were obese [5]. The National Health and Nutrition Examination Survey also reported almost a 40% obesity prevalence in the year 2015 to 2016 [6]. What is more, according to China’s national prevalence estimates from 2015 to 2019, based on Chinese criteria, there were 52.2% overweight and 27.9% obese [7]. Further, being overweight and obesity caused 11.1% of noncommunicable diseases (NCDs) in 2019. To properly treat obesity is therefore a public health imperative. Moreover, in nearly all cases of endogenous Cushing’s syndrome (CS), a condition sharing symptoms with obesity, patients who underwent surgical removal of the tumor in the ACTH-secreting pituitary adenomas experienced weight loss [8,9,10]. With the shared aim of weight loss, exercise and surgical intervention modalities have been used to curb the obesity epidemic [11,12,13,14,15], but there is little evidence regarding the comparisons among these obesity intervention protocols, especially in the lipidomic aspect [16,17,18,19,20,21].

Lipids play vital roles in both physiological and pathological processes, and they play roles in lipid trafficking, energy storage, and signal transduction [22,23,24]. Lipid metabolism and lipid homeostasis represent a typical readout parameter for systemic effects and health status. Consequently, the lipid metabolism dysfunction is reported to induce metabolic diseases, including cardiovascular diseases [25], neurological diseases [26], and even cancer [27]. Therefore, lipid profiles and correlations between lipid species and clinical outcomes are important for understanding diseases deeply. Lipidomics is a freshly emerged inter-discipline from the early 2000s, which relies on cellular lipid species based on analytical chemistry principles and technological tools. Lipidomics has become a pivotal tool for understanding a variety of scientific questions [28]. Lipidomic studies provided deep pathophysiologic insights and lipidomic biomarker signatures for obesity and weight loss interventions [28,29,30]. Here, we attempted to identify novel blood-based lipidomic biomarkers of response among different weight loss intervention methods, including exercise and two surgical interventions.

## 2. Materials and Methods

### 2.1. Demographic Characteristics

The exercise intervention cohort (Exercise) enrolled 25 volunteers (8 f; 17 m) between 9 and 16 years old. All participants had signed written informed consent forms, and this study was approved by the Ethics Committee of the Shanghai University of Sport (No. 102772021RT112). All participants participated in a 4-week high-intensity exercise intervention. According to the criteria [31,32], only participants who lost at least 3 kg during the 4-week exercise intervention were considered for this study. Blood samples were processed immediately for plasma and stored at −80 °C at baseline and 4-week follow-up.

The surgical weight loss study (LSG) enrolled a total of 36 participants (17 f; 19 m) between 19 and 61 years old who underwent laparoscopic sleeve gastrectomy (LSG). This study was approved by the Medical Ethics Committee of Huashan Hospital of Fudan University (No. 2020727). Only participants who lost at least 3 kg after surgery were considered for this study. All participants signed written informed consent forms. Blood samples were collected and immediately processed for plasma; for this study, baseline and 6-month follow-up plasma samples were used.

The Cushing study (Cushing) enrolled a total of 25 participants (25 f) between 16 and 61 years old who underwent surgical removal of the ACTH-secreting pituitary adenomas as a treatment for Cushing’s syndrome. This study was approved by the Medical Ethics Committee of Huashan Hospital of Fudan University (No. 2022886). Only participants who lost at least 3 kg after surgery were considered for this study; written informed consents were obtained and blood samples were collected from all participants before and after the surgical intervention.

All participants in this study followed the proportion of dietary energy contribution from carbohydrate–fat–protein as 50–65%:15–20%:20–30% [33,34]; follow-up visits by phone were conducted to make sure that the diet was basically consistent before and after interventions for each individual.

### 2.2. Lipid Extraction and LC-MS Analyses

Plasma samples were thawed on ice after storing at −80 °C until lipid extraction was performed. We used a modification of the MTBE-based protocol to extract lipids from plasma samples as follows: Step 1: Plasma samples (50 ul) were added to a 15 mL glass tube that had first been resolubilized in 0.5 mL HPLC grade MTBE. Tubes were vortexed for 1 min at room temperature after the addition of (i) 350 ul HPLC grade water, (ii) 1.5 mL 80% methanol pre-cooling in the refrigerator at −80 °C, and (iii) 5 mL HPLC grade MTBE, followed by mixing with a rotational table for 1 h. Step 2: 1.25 mL HPLC grade water was added to a 15 mL glass tube. Tubes were vortexed for 1 min at room temperature followed by 10 min, 1000× *g* centrifugation at 4 °C. Step 3: The upper organic phases were combined, and a nitrogen-blowing instrument was used to dry the phases overnight, and then they were stored at −80 °C. Step 4: Samples were tested with SCIEX 5500+ under an untargeted and quantitative mass spectrometry (LC-MS/MS) method, and LipidSearch (Thermo Fisher Scientific, Shanghai, China) was used to peak picking.

### 2.3. Statistical Analysis

Data processing was completed using R software version 4.2.0 [35]. Using the “stats” package in R software, the Wilcoxon Signed Rank Test was used to identify significant differences in clinical characteristics and the relative abundances of each lipid species for all samples were paired. We calculated the significantly changed lipid species within a specific intervention study, and then compared and identified the common lipid species that occurred in the three studies. The Bonferroni corrected significance threshold was used to reduce Type I error when making multiple comparisons within the data by taking the significance threshold and dividing it by the number of comparisons.

Partial least squares discriminant analysis (PLSDA) was performed on all lipids or specific lipid species of the indicated classes. PLSDA plots were generated using SIMCA (Version 14.1.0.2047, Umetrics, Sweden). Lipids relative abundance data were analyzed using the “limma” package and visualized using the “ggplot2” package as well as the “pheatmap” package in R software (https://www.r-project.org/) (accessed on 3 March 2023). The correlations of lipids and clinical characteristics were conducted using Spearman correlation coefficient to determine a significant correlation. Linear regression analyses were performed on continuous outcomes. A univariate linear regression model was used for the association between percentage change of clinical variables with lipids species levels. Logarithmic transformation of lipid data was conducted prior to analysis. Lipid data were log10-transformed (when necessary) before statistical analysis. Covariates used included age and gender. Results were presented as beta coefficients with 95% CI for linear regressions. *p*-values were corrected for multiple comparisons (Benjamini–Hochberg method), with *p* < 0.05 considered as significant.

Area under the receiver operating characteristic (AUROC) curve was used in this study to determine the model performance by using the lipid species to predict outcome groups. The larger the AUROC, the more accurate the model.

## 3. Results

### 3.1. Study Populations

Three study cohorts were included in this study: the exercise intervention cohort (Exercise), the laparoscopic sleeve gastrectomy surgical weight loss study (LSG), and the Cushing study (Cushing). Table 1 describes the clinical characteristics of the three cohorts. Standard blood analyses were performed. Briefly, a total of 25 participants with a mean age of 12.8 ± 0.36 years were recruited to the Exercise study, a total of 36 participants with a mean age of 32.64 ± 1.77 years were recruited to the LSG study, and a total of 25 participants with a mean age of 39.84 ± 2.33 years were recruited to the Cushing study. The Exercise study, the LSG study, and the Cushing study had 1, 4, and 3 participants, respectively, who could be regarded as diabetic under the diagnosis criteria [36].

Their BMIs were 29.87 ± 0.81, 38.9 ± 0.89, and 25.28 ± 0.55, respectively, at baseline. Additionally, they became 27.32 ± 0.74, 28.69 ± 0.76, and 22.81 ± 0.54, respectively, after exercise or surgical intervention. The body weights of participants in the three studies significantly decreased. Additionally, there was a largely significant decrease in patients’ clinical characteristics such as HbA1c, triglycerides, total cholesterol, and free fatty acids. Particularly, HbA1c values, which were regarded as the gold standard for predicting glycaemia-associated risks for the microvascular and macrovascular complications of diabetes mellitus over 5–10 years, were 5.74 ± 0.07, 6.12 ± 0.16, and 6.15 ± 0.15, respectively, at baseline, and changed to 5.32 ± 0.06, 5.52 ± 0.1, and 5.69 ± 0.12, respectively, after exercise or surgical intervention. The significantly decreased HbA1c in the three studies indicated that exercise and surgical interventions could improve glucose homeostasis.

### 3.2. Overview of Lipidomic Profiling

**Figure 1:**
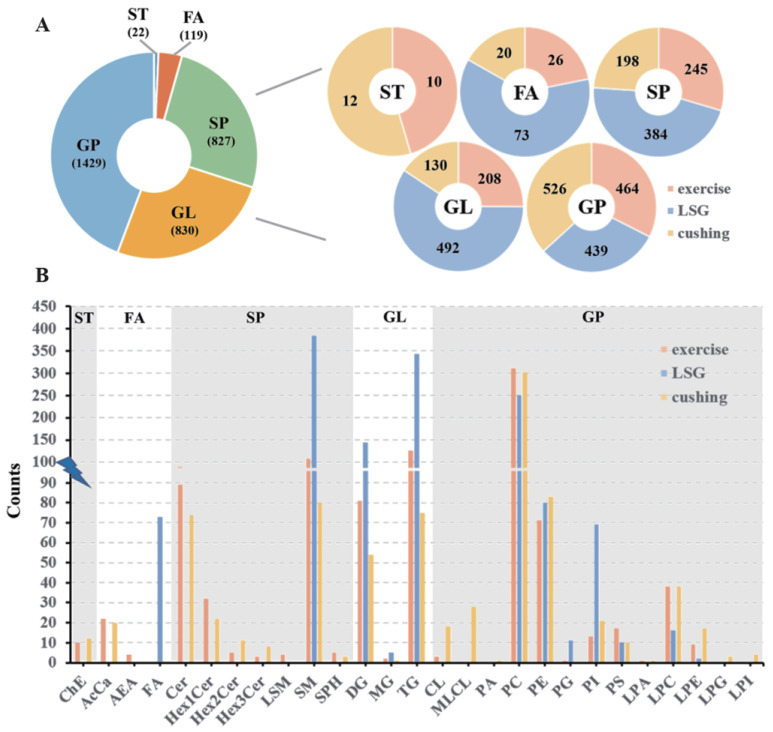
Non-targeted lipid classes. The exercise study was shown in orange, the LSG study was shown in blue, and the Cushing study was shown in yellow. (**A**) Pie charts depicting the counts of each lipid superclass. (**B**) Bar plot indicating the counts of each lipid subclass. ST: sterol lipids. FA: fatty acyls. SP: sphingolipids. GL: glycerolipids. GP: glycerophospholipids. ChE: cholesterol ester. AcCa: acylcarnitine. AEA: anandamide. FA: fatty acids. Cer: ceramide. Hex1Cer: monohexosylceramide. Hex2Cer: dihexosylceramide. Hex3Cer: trihexosylcermide. LSM: lysosphingomyelin. SM: sphingomyelin. SPH: sphingosine phosphate. DG: diacylglycerol. MG: monoglyceride. TG: triacylglycerol. CL: cardiolipin. MLCL: monolysocardiolipin. PA: phosphatidic acid. PC: phosphatidylcholine. PE: phosphatidylethanolamine. PG: phosphatidylglycerol. PI: phosphatidylinositol. PS: phosphatidylserine. LPA: lysophosphatidic acids. LPC: lysophosphatidylcholine. LPE: lysophosphatidylethanolamine. LPG: lysophosphatidylglycerol. LPI: lysophosphatidylinositol.

After data preprocessing and analysis, we quantified 953 lipid species across 22 classes for Exercise, 1388 lipid species across 12 classes for LSG, and 886 lipid species across 23 classes for Cushing, separately (Figure 1). Only a small portion of the sterol lipids (ST) and fatty acyls (FA) lipid species were quantified. Sphingolipids (SP), glycerolipids (GL) and glycerophospholipids (GP) made up a massive portion of the quantified lipid species (Figure 1A). Figure 1B shows the counts of superclass and subclass taxonomic identifications for the lipid species of each study. Diacylglycerol (DG) contains 81 lipid species for the Exercise study, 144 lipid species for the LSG study, and 54 lipid species for the Cushing study. Triacylglycerol (TG) contains 125 lipid species for the Exercise study, 343 lipid species for the LSG study, and 75 lipid species for the Cushing study. Phosphatidylcholine (PC) contains 311 lipid species for the Exercise study, 251 lipid species for the LSG study, and 302 lipid species for the Cushing study. Phosphatidylethanolamine (PE) contains 71 lipid species for the Exercise study, 80 lipid species for the LSG study, and 83 lipid species for the Cushing study. Phosphatidylinositol (PI) contains 13 lipid species for the Exercise study, 69 lipid species for the LSG study, and 21 lipid species for the Cushing study. Phosphatidylserine (PS) contains 17 lipid species for the Exercise study, 10 lipid species for the LSG study, and 10 lipid species for the Cushing study. Lysophosphatidylcholine (LPC) contains 38 lipid species for the Exercise study, 16 lipid species for the LSG study, and 38 lipid species for the Cushing study. Sphingomyelin (SM) contains 107 lipid species for the Exercise study, 384 lipid species for the LSG study, and 80 lipid species for the Cushing study. All of the eight lipid subclasses were quantified with at least 10 lipid species in each study and were therefore further analyzed.

**Figure 2:**
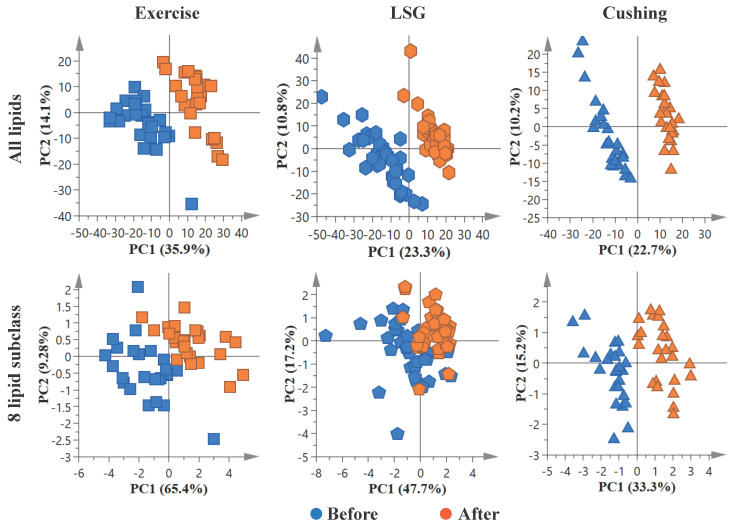
Partial least squares discriminant analysis (PLSDA) analysis. PLSDA analysis of all lipids, and eight lipid subclasses: DG, TG, PC, PE, PI, PS, LPC, and SM.

The PLSDA analysis of all lipids and DG, TG, PC, PE, PI, PS, LPC, and SM lipid subclasses demonstrated a clear separation of exercise and surgical interventions (Figure 2). Individual DG, TG, PC, PE, PI, PS, LPC, and SM species also showed changes in three studies (Figure S1). The variable importance in projection scores (VIP) summarized the importance of each lipid subclass. The larger value indicated a greater prominence; LPC was the most important in distinguishing the exercise intervention and LSG surgery, and PS was the most important in distinguishing Cushing surgery (Table S1).

**Figure 3:**
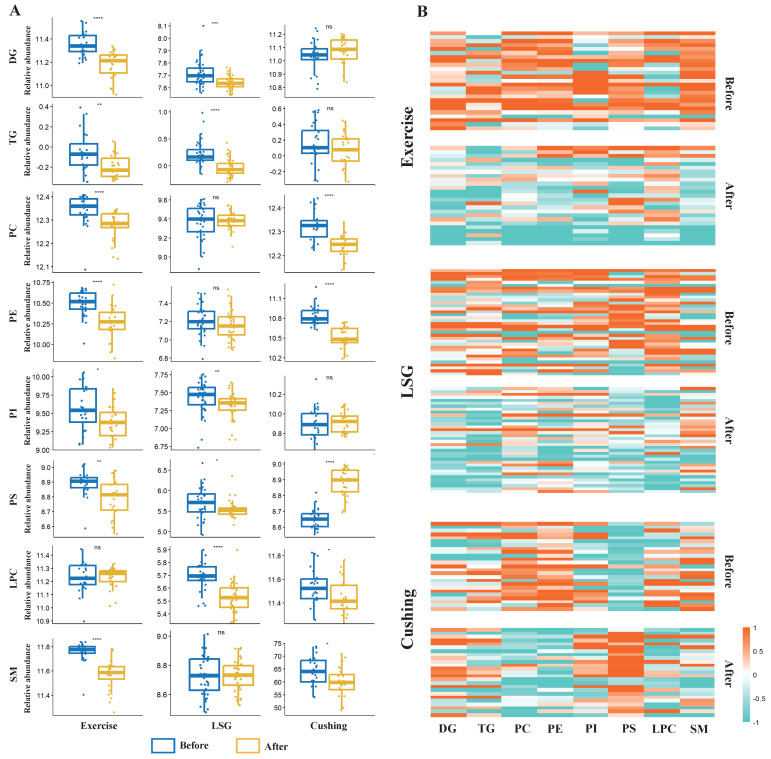
Changes in lipid subclasses. (**A**) Boxplot of relative concentrations of eight lipid subclasses which show significant differences between exercise or surgical interventions. Wilcoxon Signed Rank Test, ns: *p* > 0.05, *: *p* ≤ 0.05, **: *p* ≤ 0.01, ***: *p* ≤ 0.001, ****: *p* ≤ 0.0001. (**B**) Heatmap of relative concentrations of eight lipid subclasses.

The boxplot and heatmap revealed relative concentrations and changes of eight lipid subclasses before and after exercise or surgical interventions (Figure 3). DG and PS levels changed in all three studies after interventions. Additionally, PS was significantly decreased in Exercise and LSG while it was increased in Cushing, which indicated that although they shared the same symptom of weight loss, the three modalities had their own specialty in lipidomic metabolism. The Exercise intervention decreased all eight lipid subclasses, and the eight lipid subclasses were mainly significantly reduced, while PC exhibited a slight increase in LSG. Lipid subclasses in the Cushing study changed differently from the other two interventions, in that PS was significantly increased after surgery, which exhibited that as a traditional treatment of Cushing’s syndrome, removing the ACTH-secreting pituitary adenomas will lead to weight loss but not to a decrease in all eight lipid subclasses. PS, known as a promising candidate for treating memory loss [37], was reported to be negatively associated with disturbed sleep patterns of Parkinson’s disease patients [38], which indicated that the increase in PS might be beneficial for treating nervous disease.

### 3.3. Lipid Subclasses with Significant Relationships to BMI, HbA1c, Triglyceride, and Total Cholesterol

**Figure 4:**
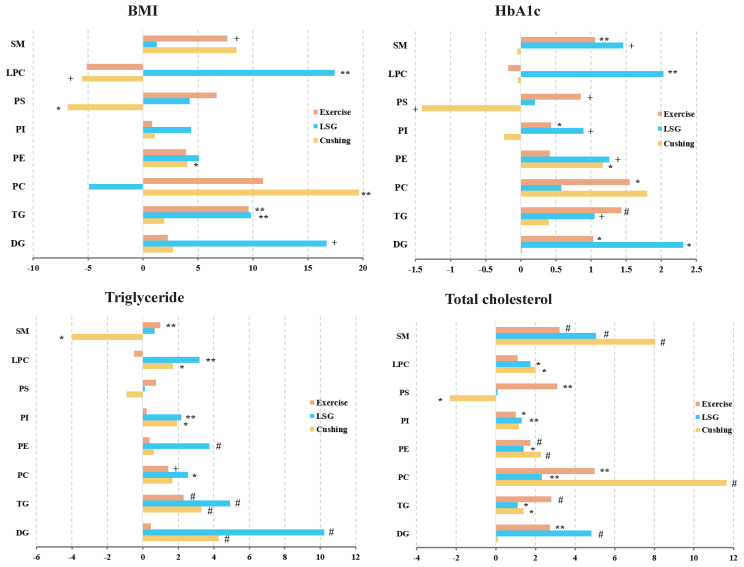
BMI, HbA1c, triglyceride, and total cholesterol were associated with lipidome. Percentage changes in total lipid species with increasing percent of BMI, HbA1c, triglyceride, and total cholesterol. Values calculated by linear regression analysis after logarithmic transformation of lipid data, + *p* < 0.1, * *p* ≤ 0.05, ** *p* ≤ 0.01, # *p* ≤ 0.001.

The percentage increase in BMI, HbA1c, triglyceride, and total cholesterol was calculated by linear regression after lipid data were logarithmically transformed (Figure 4). As for the Exercise and LSG study, the total DG, TG, PE, PI, PS, and SM changes mirrored the increases observed with increasing BMI, HbA1c, triglyceride, and total cholesterol, which can be further validated in Figure S2. Notably, after Cushing surgery, LPC tended to increase while weight loss occurred. Similarly, PC was slightly increased while BMI was decreased after bariatric surgery. However, PS was increased while BMI, HbA1c, triglyceride, and total cholesterol were decreased after Cushing surgery, and SM was increased while triglyceride was decreased.

### 3.4. Exercise and Surgical Interventions Induce Marked Changes in the Lipidome

**Figure 5:**
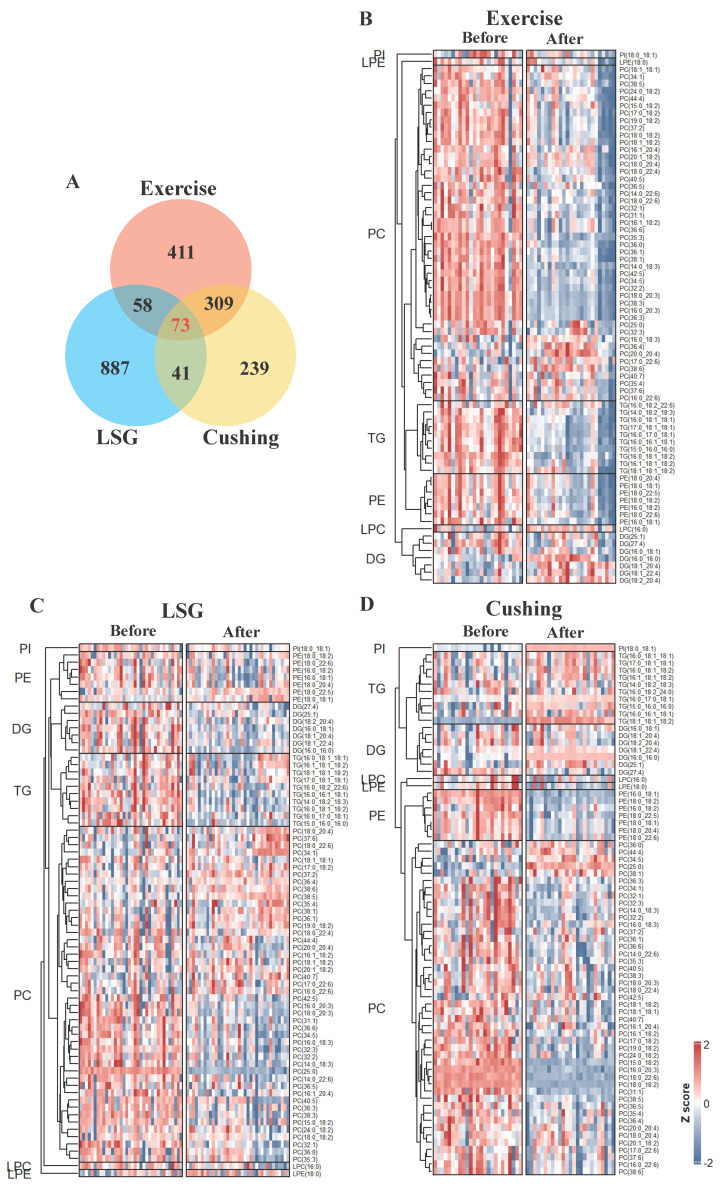
Changes in common lipid species of Exercise, LSG, and Cushing. (**A**) Venn plot shows overlap lipid species among Exercise (pink), LSG (blue), and Cushing (yellow), the Wilcoxon Signed Rank Test (*q* value < 0.1), 73 common lipid species can be found in the three studies. (**B**–**D**) Heatmap of the relative concentrations of 73 lipid species in Exercise (**B**), LSG (**C**), and Cushing (**D**).

The Wilcoxon Signed Rank Test was used to calculate significantly changed lipid species (*q* value < 0.1). We found significant changes in lipid species under weight loss interventions (Figure S3). A set of 73 lipid species were shared among three studies, which may be highly correlated with weight loss regardless of the intervention methods (Figure 5A). A total of 46 out of 73 lipid species were PC, and 10 out of 73 lipid species were TG. The *Z*-score heatmap in Figure 5B–D showed relative concentrations of the 73 lipid species in Exercise, LSG, and Cushing separately.

The clustering was by lipid subclasses horizontally, and most lipid species tended to decrease after the exercise intervention (Figure 5B), while PC(20:0_20:4), PC(16:0_22:6), DG(16:0_16:0), DG(18:1_20:4), DG(18:1_22:4), DG(18:2_20:4), PC(38:6), PC(17:0_22:6), and LPC(16:0) were increased after exercise. After laparoscopic sleeve gastrectomy, lipid species changed differently from exercise intervention in that PC(16:0_22:6), PC(17:0_18:2), PC(17:0_22:6), PC(18:0_20:4), PC(18:0_22:6), PC(18:1_18:2), PC(19:0_18:2), PC(20:1_18:2), PC(34:1), PC(35:4), PC(36:1), PC(36:4), PC(37:2), PC(37:6), PC(38:1), PC(38:5), PC(38:6), PC(40:7), LPE(18:0), PE(18:0_18:1), and PE(18:0_22:5) were increased, while the other lipid species were decreased. After Cushing surgery, TG(15:0_16:0_16:0), TG(16:0_16:1_18:1), TG(16:0_17:0_18:1), TG(16:0_18:1_18:1), TG(16:0_18:1_18:2), TG(16:1_18:1_18:2), TG(17:0_18:1_18:1), TG(18:1_18:1_18:2), DG(16:0_16:0), DG(16:0_18:1), DG(18:1_22:4), DG(25:1), PI(18:0_18:1), PC(25:0), PC(34:5), PC(36:0), PC(38:1), PC(42:5), and PC(44:4) were increased while the other lipid species were decreased.

Surprisingly, PC(36:4), PC(38:6), and PC(17:0_22:6) were all significantly increased in the Exercise and LSG studies, and the other 46 of 73 lipid species were decreased in the two studies. Of them, for example, PC(36:4) were reported to be associated with decreased risk of diabetes [39], and were reported to be decreased in gastric cancer tissues [40], which told us that both exercise and bariatric surgery induced several similar lipid species changes that deserved further analysis. As for surgical intervention, PC(38:1) was both increased in the LSG and Cushing studies, but 20 of 21 lipid species were increased in LSG but decreased in the Cushing study (Figure 5C,D), which indicated that with the shared symptom of weight loss, different interventions triggered various lipidomic responses. What is more, 30 of 73 lipid species were decreased in all three studies, including DG(27:4), PC(38:3), PE(16:0_18:2), PE(16:0_18:2), PE(18:0_18:2), and PC(16:1_18:2), which warrants further analysis and research as some of those lipid species may act as potential weight loss biomarkers regardless of the weight loss interventions used.

### 3.5. Correlations between Lipid Species and Clinical Characteristics

**Figure 6:**
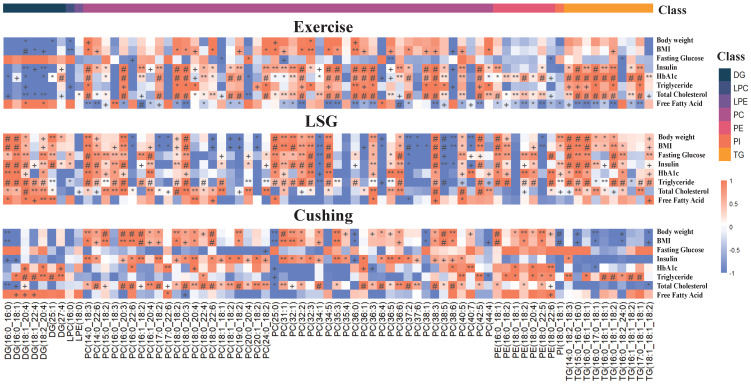
Correlations between lipid species and clinical characteristics in Exercise, LSG, and Cushing. Spearman correlations computed between lipid species and clinical features, + *p* < 0.1, * *p* < 0.05, ** *p* < 0.01, # *p* < 0.001. Red indicated positive correlations and blue indicated negative correlations.

The pairwise relationship between 73 lipid species and clinical characteristics was visualized in Figure 6, based on the Spearman correlation coefficient, implying a high correlation for most clinical variables with lipid species. PC(14:0_18:3), for example, was positively correlated with body weight and BMI in three studies, and was significantly correlated with fasting insulin and total cholesterol in all studies. PC(36:6) was also positively correlated with fasting insulin and total cholesterol in all studies. However, PC(38:5) was positively correlated with fasting insulin, HbA1c, total glyceride, and total cholesterol in Exercise and Cushing, but negatively correlated in LSG, which indicated that LSG might trigger the downregulation of PC(38:5) compared to Exercise and the Cushing surgical intervention. Likewise, DG(18:1_20:4) and DG(18:2_20:4) were negatively correlated with most of the clinical variables in Exercise, while being positively correlated in LSG, which demonstrates that different weight loss interventions result in different lipidomic signatures. However, several lipid species exist that can be used to indicate weight loss, regardless of intervention modalities.

### 3.6. Utility of Lipid Species as Biomarkers of Weight Loss

**Figure 7:**
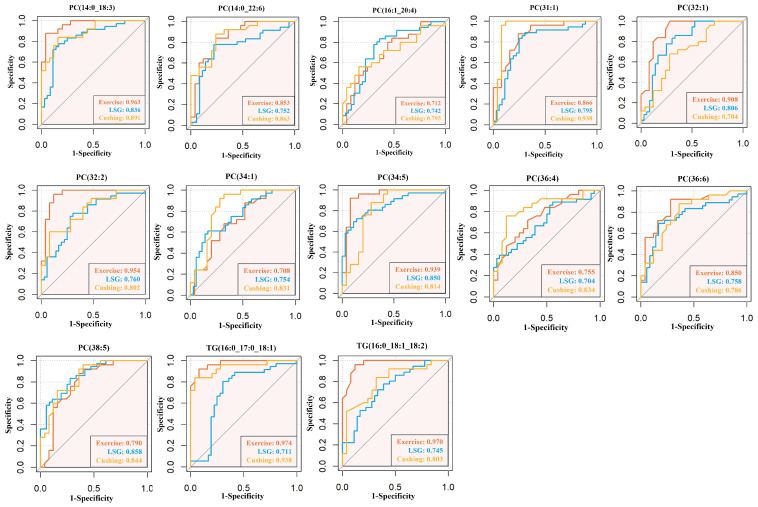
The ROC curves of predictive models generated by lipid species. AUC scores of ROC curves are listed on the right of the prediction accuracy.

We further investigated the power of lipid species to predict weight loss situations. The AUROC of all 73 common lipid species in 3 studies was calculated. The top 13 most predictive lipid species among the 3 studies were selected and visualized in Figure 7, and the AUC scores showed diagnostic accuracy for prediction. PC(14:0_18:3) showed the highest diagnostic accuracy in Exercise (AUROC = 0.963), LSG (AUROC = 0.836), and Cushing (AUROC = 0.891). PC(14:0_22:6), PC(16:1_20:4), PC(31:1), PC(32:1), PC(32:2), PC(34:1), PC(34:5), PC(36:4), PC(36:6), PC(38:5), TG(16:0_17:0_18:1), and TG(16:0_18:1_18:2) showed good predictive accuracy in diagnosing the weight loss situation in Exercise, LSG, and Cushing (AUROC > 0.7). Of them, PC(32:1), PC(32:2), and PC(36:6) were reported to be significantly decreased after weight loss [41], which was in accordance with our results.

**Figure 8:**
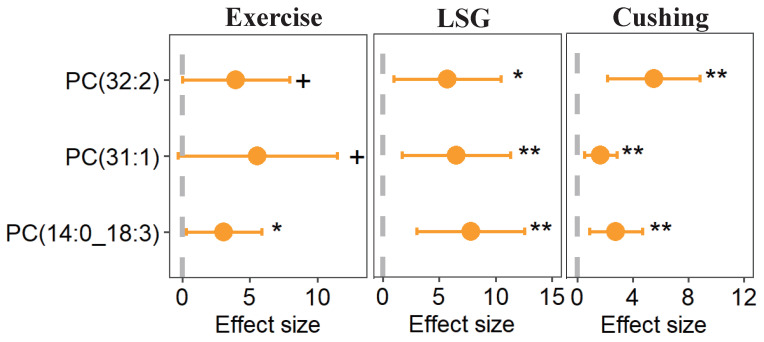
Potential biomarkers of weight loss. Values calculated by linear regression analysis after logarithmic transformation of lipid data, + *p* < 0.1, * *p* ≤ 0.05, ** *p* ≤ 0.01.

Moreover, we identified three lipid species that were highly correlated with weight loss, and which showed significant concurrent changes in three weight loss interventions (Figure 8). Calculated by linear regression, PC(14:0_18:3), PC(31:1), and PC(32:2) were significantly associated with BMI changes in three studies, which indicated that these three lipid species might act as lipidomic markers for weight loss interventions, regardless of the modalities used.

## 4. Discussion

By comparing the lipid changes of three weight loss intervention cohorts, we have identified lipid subclasses and lipid species that are associated with decreased BMI and improvements in HbA1c, total glyceride, and total cholesterol. Specifically, we found that decreases in DG, TG, PE, PI, PS, and SM levels were associated with weight loss interventions. Meanwhile, increased concentrations of several species of PE and PI were detected in NASH subjects [42]. More importantly, we identified that the Exercise intervention caused a large decrease in SM, PC, and TG. Our findings are in agreement with previous studies; some lipid species of SM were reported to be positively associated with type 2 diabetes [43] (T2D), and decreases in SM (SM(16:0), SM(16:1), SM(18:2)) were reported to be associated with lower BMI and T2D risk [44]. Furthermore, higher levels of TG were observed in increased body weight and T2D [45], and which can be further validated in the Framingham Heart Study [46]. PC is one of the main components of the surface layer of lipoproteins, which was found to be associated with progression to T2D [47]. In addition, we have demonstrated that bariatric surgery decreases most lipid species of DG, TG, PI, and PE. The Cushing surgery (to remove the tumor in the pituitary) induced higher PS and SM levels together with weight loss. PS, in particular, has been reported to regulate a variety of neuroendocrine responses, including the release of acetylcholine, dopamine, and noradrenaline [48]. What is more, PS was used for treating memory loss and was beneficial for the treatment of some nervous diseases, which indicated that with the same symptom of weight loss, the Cushing surgery induced a significant increase in PS that might be good for the nervous system.

In agreement with several recent studies, in children with substantial weight loss undergoing a 1-year lifestyle intervention, the LPC(18:1), LPC(18:2), and LPC(20:4) increased significantly [49]. Additionally, LPC(16:0) was reported to be reduced in patients with acute liver failure, and was highly associated with a poor prognosis [50]. LPC(16:0) was also reported to be negatively correlated with BMI in obese subjects compared with control subjects [51]. Particularly, LPC(16:0) was significantly increased after exercise intervention, but was decreased in LSG and Cushing studies, which demonstrated that exercise may act as well as surgical interventions in improving weight loss lipidomic metabolism.

As previous studies showed, PC(32:1) and PC(38:3) were reported to be positively correlated with BMI [52,53], and were also reported to be positively correlated with obesity [54]. PC(40:5) were reported to be positively correlated with BMI, which was in coincidence with the relationship in the Exercise study and the LSG study [53]. Additionally, PC(34:1), PC(38:5), PC(38:6), LPC(16:0), and LPE(18:0) were reported to be negatively correlated with BMI [53,55]. PC(44:4) was reported to be negatively correlated with BMI [52]. PC(18:1_18:1) was negatively correlated with BMI [56].

More significantly, in this study, PC(14:0_18:3) showed the highest diagnostic accuracy in Exercise (AUROC = 0.963), LSG (AUROC = 0.836), and Cushing (AUROC = 0.891) interventions. PC(31:1) showed better diagnostic accuracy in Exercise (AUROC = 0.908), LSG (AUROC = 0.806), and Cushing (AUROC = 0.704). Additionally, PC(32:2) also showed better diagnostic accuracy in Exercise (AUROC = 0.954), LSG (AUROC = 0.760), and Cushing (AUROC = 0.802). According to previous studies, PC(31:1) was highly expressed in liver cancer metastatic lesions [57], and weight loss induced a decrease in PC(32:2) together with a decrease in total fat after 8 weeks of a low-calorie diet (LCD) [41]. PC(32:2) was also reported to be decreased and aligned with lower levels of total cholesterol and low-density lipoprotein cholesterol after one year of a low-calorie diet in the Satiety Innovation (SATIN) study [58].

What is more, PC(32:2) was observed to be positively correlated with BMI after a one-year lifestyle intervention, and they also found that lower concentrations of long chain unsaturated phosphatidylcholines (PC) were significant predictors of BMI reduction [59]. Additionally, PC(32:2) was reported to be positively associated with BMI [52], and was also observed to be positively correlated with obesity compared to the normal weight group [54]. The three lipid species were identified in this study to be highly associated with weight loss in three studies; taken together, these results suggest a potential predictive value for PC(14:0_18:3), PC(31:1), and PC(32:2) to act as potential lipidomic biomarkers for weight loss interventions.

## 5. Conclusions

In this study, we presented the lipidomic data of three weight loss interventions, and found that although under different methods, there were three lipid species that were highly associated with weight loss. The potential value of PC(14:0_18:3), PC(31:1), and PC(32:2), which act as potential lipidomic biomarkers, is demonstrated, and this information can be further used in personalized approaches studies involving weight loss interventions.

## Figures and Tables

**Table 1:** Baseline characteristics of the study populations ^1^.

Clinical Characteristics	Exercise			LSG			Cushing		
	Before	After	*p* Value ^2^	Before	After	*p* Value	Before	After	*p* Value
N	25			36			25		
female (%)	32%			47.2%			100%		
age (years)	12.8 ± 0.36			32.64 ± 1.77			39.84 ± 2.33		
Body Weight (kg)	83.61 ± 3.98	75.12 ± 3.58	***	112.53 ± 3.00	83.13 ± 2.41	***	65.67 ± 2.15	58.79 ± 1.92	*
BMI (kg/m^2^)	29.87 ± 0.81	27.32 ± 0.74	*	38.9 ± 0.89	28.69 ± 0.76	***	25.28 ± 0.55	22.81 ± 0.54	**
FBG (mmol/L)	4.16 ± 0.12	4.23 ± 0.07		5.61 ± 0.18	4.86 ± 0.16	***	5.43 ± 0.24	4.94 ± 0.14	
Insulin (pmol/L)	12.48 ± 1.14	7.1 ± 0.63	***	24.87 ± 2.80	9.88 ± 1.10	***	17.52 ± 2.60	9.96 ± 1.65	**
HbA1c (%)	5.74 ± 0.07	5.32 ± 0.06	***	6.12 ± 0.16	5.52 ± 0.10	***	6.15 ± 0.15	5.69 ± 0.12	*
HDL (mmol/L)	1.29 ± 0.06	1.14 ± 0.04		1.01 ± 0.03	1.16 ± 0.04	**	1.5 ± 0.07	1.2 ± 0.04	***
LDL (mmol/L)	2.73 ± 0.12	2.02 ± 0.08	***	3.31 ± 0.16	3.1 ± 0.12		3.08 ± 0.17	2.5 ± 0.11	**
Triglyceride (mmol/L)	0.96 ± 0.10	0.66 ± 0.03	**	2.07 ± 0.28	0.99 ± 0.07	***	1.58 ± 0.21	1.30 ± 0.13	
Total Cholesterol (mmol/L)	4.43 ± 0.16	3.41 ± 0.10	***	4.96 ± 0.17	4.73 ± 0.13		4.94 ± 0.30	4.21 ± 0.15	**
Free Fatty Acids (mmol/L)	0.89 ± 0.05	1.16 ± 0.07	**	0.62 ± 0.04	0.51 ± 0.03	*	0.460 ± 0.03	0.55 ± 0.022	*

^1^ BMI: body mass index. FBG: fasting blood glucose. HbA1c: glycated hemoglobin. HDL: high-density lipoprotein (HDL) cholesterol (mmol/L). LDL: low-density lipoprotein (LDL) cholesterol (mmol/L). Continuous variables are listed as mean ± standard error. “kg” is kilograms. “%” is percent. ^2^ *p* Values were estimated by Wilcoxon signed rank test, * *p* < 0.05, ** *p* < 0.01, *** *p* < 0.001.

## Data Availability

The data presented in this study are available on request from the corresponding author.

## Supplementary Materials

The following supporting information can be downloaded at https://www.mdpi.com/article/10.3390/nu15071784/s1, Figure S1: Partial least squares discriminant analysis (PLSDA); Figure S2: Spearman rank correlation coefficients of class sums; Figure S3: Volcano plot of lipid species indicating lipid changes associated with weight loss interventions; Table S1: VIP scores of PLSDA analysis. Table S2: Weight loss of the three studies.

## Abbreviations

ST: Sterol Lipids; FA: Fatty acyls; SP: Sphingolipids; GL: Glycerolipids; GP: Glycerophospholipids; ChE: cholesterol ester; AcCa: Acylcarnitine; AEA: Anandamide; FA: Fatty acids; Cer: Ceramide; Hex1Cer: Monohexosylceramide; Hex2Cer: Dihexosylceramide; Hex3Cer: Trihexosylcermide; LSM: Lysosphingomyelin; SM: Sphingomyelin; SPH: Sphingosine phosphate; DG: Diacylglycerol; MG: Monoglyceride; TG: Triacylglycerol; CL: Cardiolipin; MLCL: Monolysocardiolipin; PA: Phosphatidic acid; PC: Phosphatidylcholine; PE: Phosphatidylethanolamine; PG: Phosphatidylglycerol; PI: Phosphatidylinositol; PS: Phosphatidylserine; LPA: Lysophosphatidic acids; LPC: Lysophosphatidylcholine; LPE: Lysophosphatidylethanolamine; LPG: Lysophosphatidylglycerol; LPI: Lysophosphatidylinositol.
