# Supplementary material for: Lipid Profiling Reveals Lipidomic Signatures of Weight Loss Interventions
Source: Nutrients. 2023 Apr 6;15(7):1784. doi: 10.3390/nu15071784 (PMC10097218; doi:10.3390/nu15071784)
Supplement: Supplementary file 1 [file nutrients-15-01784-s001.zip › nutrients-2291407-supplementary.pdf]

Supplementary Materials:

Supplementary Table S1. VIP scores of PLSDA analysis.

	Exercise	LSG	Cushing
LPC	1.33403	1.49417	0.537296
SM	1.20663	0.743749	0.820176
TG	1.03045	1.05002	0.966597
DG	0.949263	0.982946	0.628143
PS	0.919605	0.727666	1.65306
PC	0.868191	0.959442	1.19942
PE	0.842632	0.958827	1.24025
PI	0.701442	0.881015	0.0173637

Supplementary Table S2. Weight loss of the three studies.

	Exercise			LSG			Cushing	
ID	Body weight change	Percentage of body weight change	ID	Body weight change	Percentage of body weight change	ID	Body weight change	Percentage of body weight change
exercise_1	3.8	6	LSG_1	32	28	cushing_1	7	10
exercise_2	5.4	7	LSG_2	19	19	cushing_2	7	11
exercise_3	10.3	10	LSG_3	24	23	cushing_3	5	8
exercise_4	11	11	LSG_4	21	21	cushing_4	4	5
exercise_5	5.2	9	LSG_5	45	40	cushing_5	3	4
exercise_6	12.8	13	LSG_6	25	22	cushing_6	5	6
exercise_7	7.4	10	LSG_7	34	28	cushing_7	3	6
exercise_8	7.85	10	LSG_8	26.5	26	cushing_8	10	13
exercise_9	7.1	8	LSG_9	27	18	cushing_9	5	7
exercise_10	10.7	8	LSG_10	34	31	cushing_10	8	14
exercise_11	5.5	11	LSG_11	32	28	cushing_11	15	19
exercise_12	6.2	10	LSG_12	45	31	cushing_12	3	6
exercise_13	6.2	10	LSG_13	35.7	34	cushing_13	17.5	22
exercise_14	7.1	9	LSG_14	25.8	26	cushing_14	5	8
exercise_15	11.1	12	LSG_15	11	9	cushing_15	8	12
exercise_16	5	7	LSG_16	40	29	cushing_16	13.5	24
exercise_17	9.5	10	LSG_17	27.2	22	cushing_17	5	9
exercise_18	10.4	12	LSG_18	19.5	25	cushing_18	3.3	6
exercise_19	6.5	11	LSG_19	22	24	cushing_19	5.2	8
exercise_20	11.2	11	LSG_20	19	21	cushing_20	3.5	6
exercise_21	9.15	11	LSG_21	51	40	cushing_21	5	9
exercise_22	10.9	12	LSG_22	23	30	cushing_22	6	9
exercise_23	11.9	10	LSG_23	30	31	cushing_23	4	7
exercise_24	10.2	12	LSG_24	23	22	cushing_24	9	17
exercise_25	9.8	12	LSG_25	42	30	cushing_25	12	14
			LSG_26	31	26			
			LSG_27	26	23			
			LSG_28	39.2	28			
			LSG_29	36.4	28			
			LSG_30	19	22			
			LSG_31	20	21			
			LSG_32	33	27			
			LSG_33	44	36			
			LSG_34	20	18			
			LSG_35	26	24			
			LSG_36	30	26			

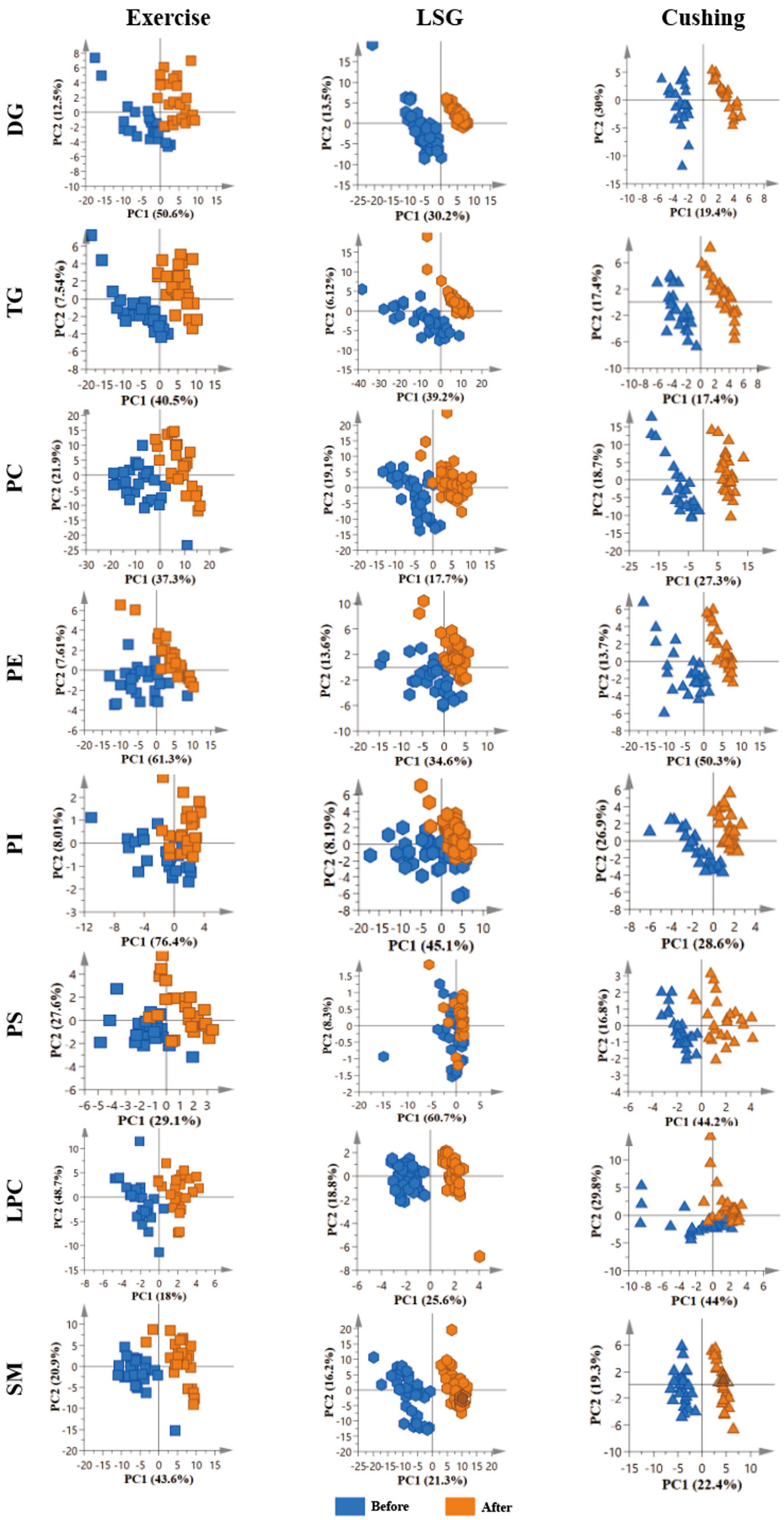

Supplementary Figure S1. Partial least squares discriminant analysis (PLSDA). Samples before intervention is marked in blue, samples after intervention is marked in orange.

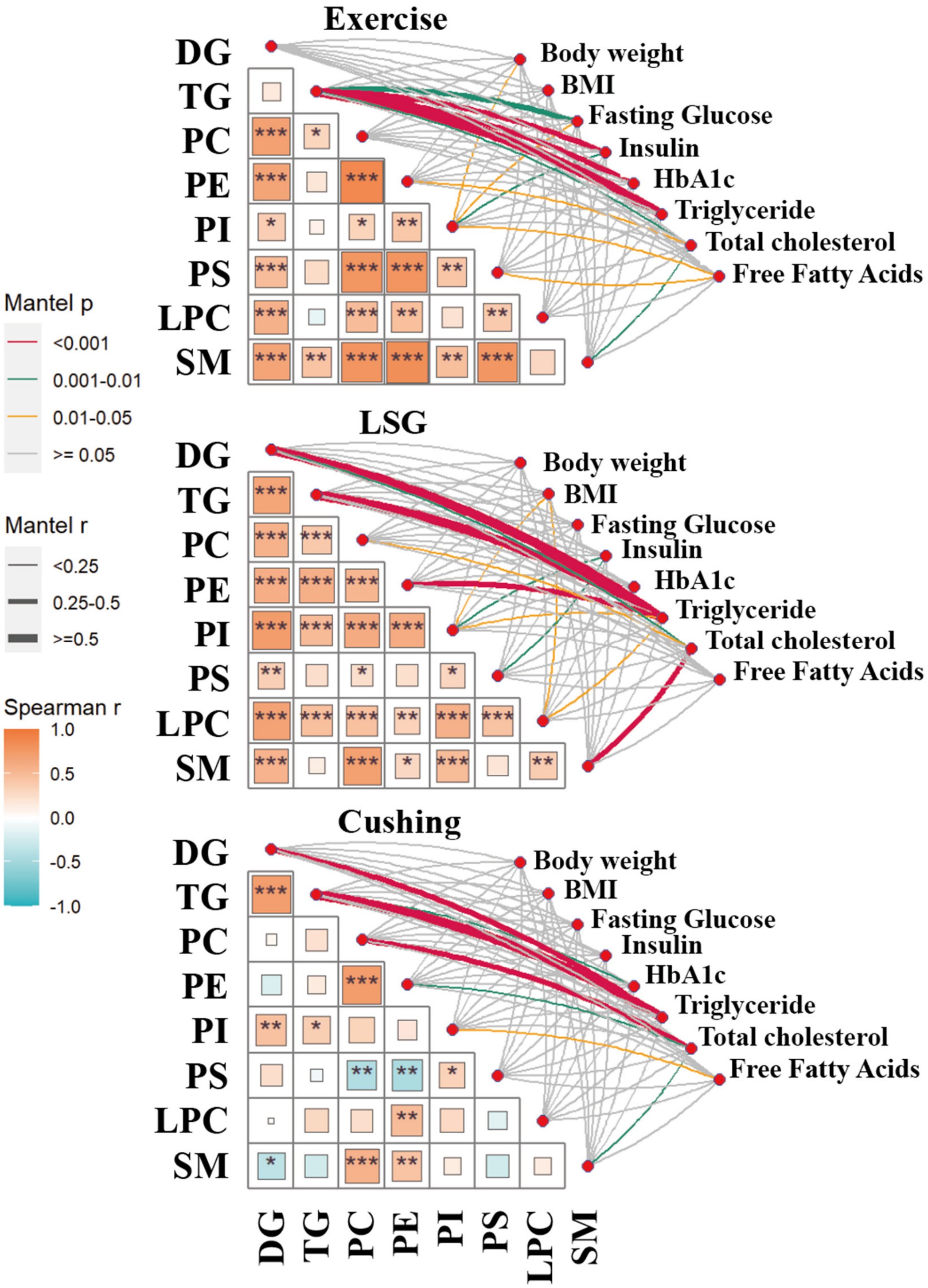

Supplementary Figure S2. Spearman rank correlation coefficients of class sums. Pairwise comparisons of 8 lipid subclasses are shown, with a color gradient denoting Spearman's correlation coefficients, *p<0.05, **p<0.01, ***p<0.001. Lipid subclasses were related to each clinical factor by partial (geographic distance–corrected) Mantel tests. Edge width corresponds to the Mantel's r statistic for the corresponding distance correlations, and edge color denotes the statistical significance based on 9,999 permutations.

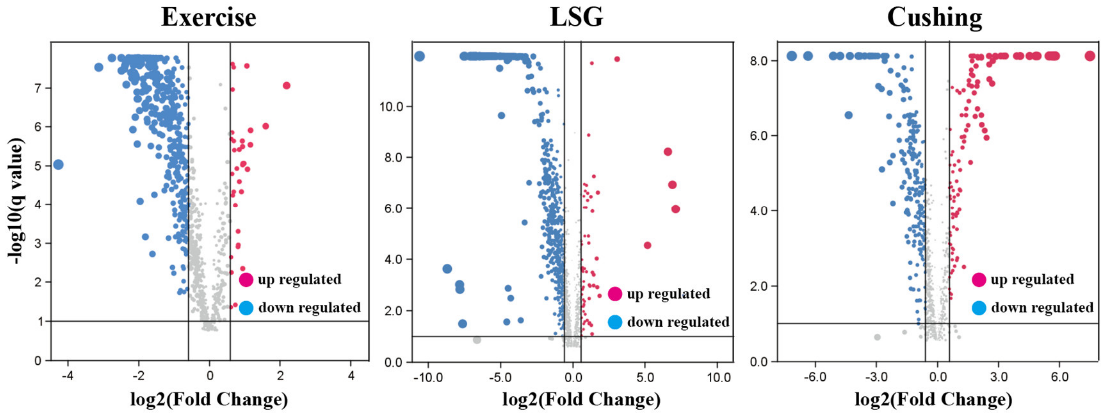

Supplementary Figure S3. Volcano plot of lipid species indicating lipid changes associated with weight loss interventions (fold change > 1.5 and q value < 0.1 by Wilcoxon Signed Rank Test, q value was calculated using the q value package based on the default settings).
